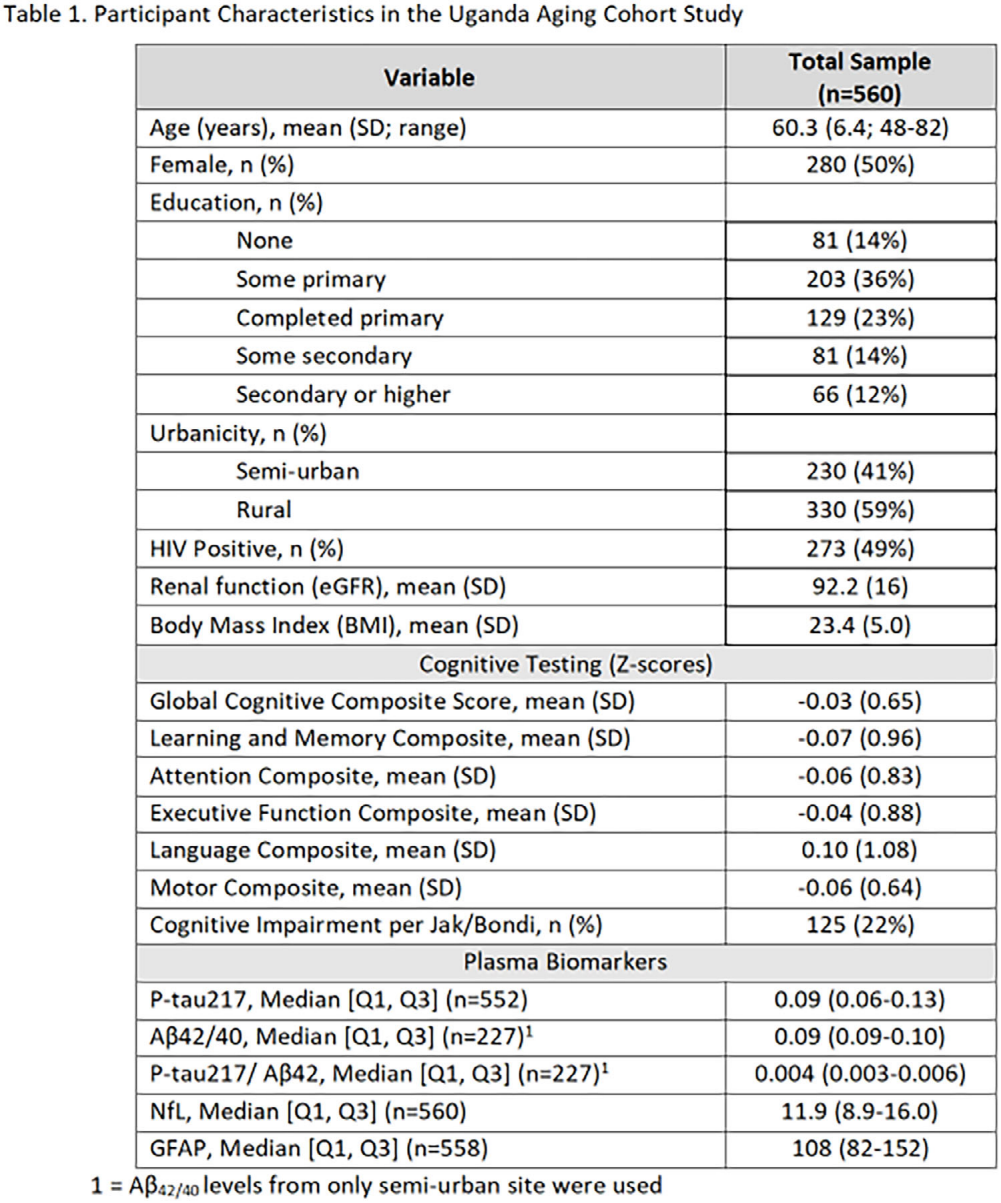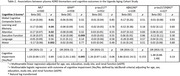# Plasma Biomarker Association with Cognition in Uganda

**DOI:** 10.1002/alz70856_106808

**Published:** 2026-01-08

**Authors:** Jeremy A. Tanner, Roslyn Valdespino, Tiffany F. Kautz, Godfrey Masette, Gabrielle Hromas, Chen‐Pin Wang, Jayandra Jung Himali, Robert Paul, Noeline Nakasujja, Zahra Reynolds, Flavia Atwine, Edna Tindimwebwa, Meredith Greene, Eliza Passell, Christine S Ritchie, Susanne S Hoeppner, Alexander C Tsai, Janet Seeley, Lawren VandeVrede, Amy Werry, Elena Tsoy, Katherine L. Possin, Josephine E Prynn, Suzanne E. Schindler, Sudha Seshadri, Samson Okello, Stephen Asiimwe, Deanna Saylor, Mark J Siedner

**Affiliations:** ^1^ University of Texas Health San Antonio, San Antonio, TX, USA; ^2^ Mbarara University of Science & Technology (MUST), Mbarara, Uganda; ^3^ University of Missouri, St Louis, MO, USA; ^4^ Makerere University, Kampala, Central, Uganda; ^5^ Massachusetts General Hospital (MGH), Boston, MA, USA; ^6^ Kabwohe Clinical Research Center (KCRC), Kabwohe, Uganda; ^7^ Indiana University School of Medicine., Indianapolis, IN, USA; ^8^ London School of Hygiene and Tropical Medicine, London, United Kingdom; ^9^ University of California San Francisco, San Francisco, CA, USA; ^10^ Global Brain Health Institute (GBHI), University of California San Francisco (UCSF); & Trinity College Dublin, San Francisco, CA, USA; ^11^ King's College London, Strand, London, United Kingdom; ^12^ Washington University in St. Louis, St. Louis, MO, USA; ^13^ University of North Carolina, Chapel Hill, NC, USA

## Abstract

**Background:**

Over 60% of individuals with ADRDs live in low‐ and middle‐income countries (LMICs). Sub‐Saharan Africa (SSA) is projected to have the fastest growth in older adults, including people living with HIV (PLWH). Plasma ADRD biomarkers are promising diagnostic tools, but studies have been limited to the Global North. We evaluated the association of plasma ADRD biomarkers with cognition in the Uganda Aging Cohort Study (UACS), a prospective cohort of PLWH on antiretroviral therapy and demographically similar HIV‐uninfected individuals recruited via population‐sampling approaches.

**Method:**

Blood samples were collected in EDTA tubes, centrifuged, aliquoted, and frozen at ‐80°C at semi‐urban (*n* = 320) and rural (*n* = 330) sites. Plasma was shipped to the UTHSA Biggs Laboratory. *p*‐tau217, Aβ42/40, and *p*‐tau217/Aβ42 were measured on Fujirebio Lumipulse, and GFAP and NfL on Quanterix Simoa. Rural site Aβ42/40 and *p*‐tau217/Aβ42 did not pass quality control due to cold‐chain storage complications and were excluded from this analysis. Cognitive evaluations used locally validated tests. Raw scores were converted to regression‐based Z‐scores and averaged at the domain and overall level. Jak/Bondi criteria defined presence/absence of cognitive impairment. Multivariable regressions assessed associations between log‐transformed plasma biomarkers and cognitive outcomes adjusting for age, sex, education, site, and eGFR. Sensitivity analyses adjusted for HIV serostatus.

**Result:**

560 total participants (49% PLWH) were included, and average age was 60.3 years (Table 1). Elevated plasma NfL was associated with worse overall cognition, learning/memory, attention, executive function, and motor performance (Table 2). Elevated plasma GFAP was associated with worse executive function and 62% higher odds of cognitive impairment in this domain. Plasma *p*‐tau217, Aβ42/40, and *p*‐tau217/Aβ42 were not associated with cognition. Results were consistent in sensitivity analyses adjusted for HIV serostatus.

**Conclusion:**

Plasma NfL and GFAP are promising biomarkers associated with cognition in adults residing in Uganda, including in PLWH. The lack of association between plasma *p*‐tau217 and cognition suggests that cognitive impairment in this younger cohort is being driven by non‐Alzheimer disease etiologies. Further studies in diverse global populations are needed to inform appropriate use of new biology‐based diagnostic criteria for AD and to identify methods that do not require sample storage to expand access to LMICs.